# Large batoid fishes frequently consume stingrays despite skeletal damage

**DOI:** 10.1098/rsos.170674

**Published:** 2017-09-06

**Authors:** Mason N. Dean, Joseph J. Bizzarro, Brett Clark, Charlie J. Underwood, Zerina Johanson

**Affiliations:** 1 Department of Biomaterials, Max Planck Institute of Colloids and Interfaces, 14424 Potsdam, Germany; 2 Institute of Marine Sciences, University of California, Santa Cruz, CA 95060, USA; 3 Fisheries Ecology Division, Southwest Fisheries Science Center, National Marine Fisheries Service, 110 McAllister Way, Santa Cruz, CA 95060, USA; 4 Core Research Laboratories, Natural History Museum, London, UK; 5 Department of Earth Sciences, Natural History Museum, London, UK; 6 Department of Earth and Planetary Sciences, Birkbeck College, Malet Street, London, UK

**Keywords:** cartilage, elasmobranch, predation, stingray spine, skeletal callus, tesserae

## Abstract

The shapes of vertebrate teeth are often used as hallmarks of diet. Here, however, we demonstrate evidence of frequent piscivory by cartilaginous fishes with pebble-like teeth that are typically associated with durophagy, the eating of hard-shelled prey. High-resolution micro-computed tomography observation of a jaw specimen from one batoid species and visual investigation of those of two additional species reveal large numbers of embedded stingray spines, arguing that stingray predation of a scale rivalling that of the largest carnivorous sharks may not be uncommon for large, predatory batoids with rounded, non-cutting dentition. Our observations demonstrate that tooth morphology is not always a reliable indicator of diet and that stingray spines are not as potent a deterrent to predation as normally believed. In addition, we show that several spines in close contact with the jaw skeleton of a wedgefish (*Rhynchobatus*) have become encased in a disorganized mineralized tissue with a distinctive ultrastructure, the first natural and unequivocal evidence of a callus-building response in the tessellated cartilage unique to elasmobranch skeletons. Our findings reveal sampling and analysis biases in vertebrate ecology, especially with regard to the role of large, predatory species, while also illustrating that large body size may provide an escape from anatomical constraints on diet (e.g. gape size, specialist dentition). Our observations inform our concepts of skeletal biology and evolution in showing that tessellated cartilage—an ancient alternative to bone—is incapable of foreign tissue resorption or of restoring damaged skeletal tissue to its original state, and attest to the value of museum and skeletal specimens as records of important aspects of animal life history.

## 1. Introduction

A long-standing paradigm in vertebrate functional morphology dictates that the gross form of a tooth attests to its function and therefore can also be used to delineate broad dietary differences [1–4]. This concept makes intuitive sense—sharp edges are effective for slicing, pointed tools for puncturing and flat surfaces for crushing—and has been largely borne out by experimental and dietary studies (e.g. [5–10]). Tooth form has therefore been considered as one possible constraint framing trophic ecology and evolution, also being used to infer dietary guilds and feeding ecology of extinct taxa (e.g. [3,11,12]).

The elasmobranch fishes (sharks, rays and relatives) have confounded the notion of an invariant, simplistic link between tooth form and diet. Although they are far less speciose, elasmobranchs possess a dental diversity that rivals that of mammals [13,14], with teeth often described by their inferred function (e.g. as ‘clutching’ or ‘tearing’ or ‘crushing’ teeth [7,15]). However, pronounced differences in cusp shape or curvature do not necessarily translate to differences in puncturing, cutting or crushing performance [14,16–18], some species eat foods that are far harder or softer than their dentitions suggest [13,19], and two species have been shown to be able to reorient their teeth to perform multiple roles not predictable by their tooth morphology [13,20].

Here, we provide evidence that an elasmobranch fish with rounded teeth (widely associated with durophagy) fed regularly on stingrays, a piscivorous diet often believed to demand pointed teeth for grasping and/or ripping (e.g. [21,22]). The observation comes from a jaw of an adult wedgefish (*Rhynchobatus* sp.) that was revealed, via high-resolution micro-computed tomography (CT) scanning, to have dozens of stingray spines embedded in its soft tissue and cartilage. This finding runs counter to the historic dogma concerning links between dental form and diet, and also the trophic spectrum of batoid fishes (rays and relatives), which was not believed to include stingrays [23,24], while also supporting an emerging viewpoint that elasmobranch diets are highly variable [25–30]. We discuss the implications of this observation for the feeding ecology and behaviour of elasmobranchs and also their skeletal biology, by providing the first unequivocal demonstration of the elasmobranch skeletal tissue's natural response to damage.

## 2. Material and methods

**Figure 1.**
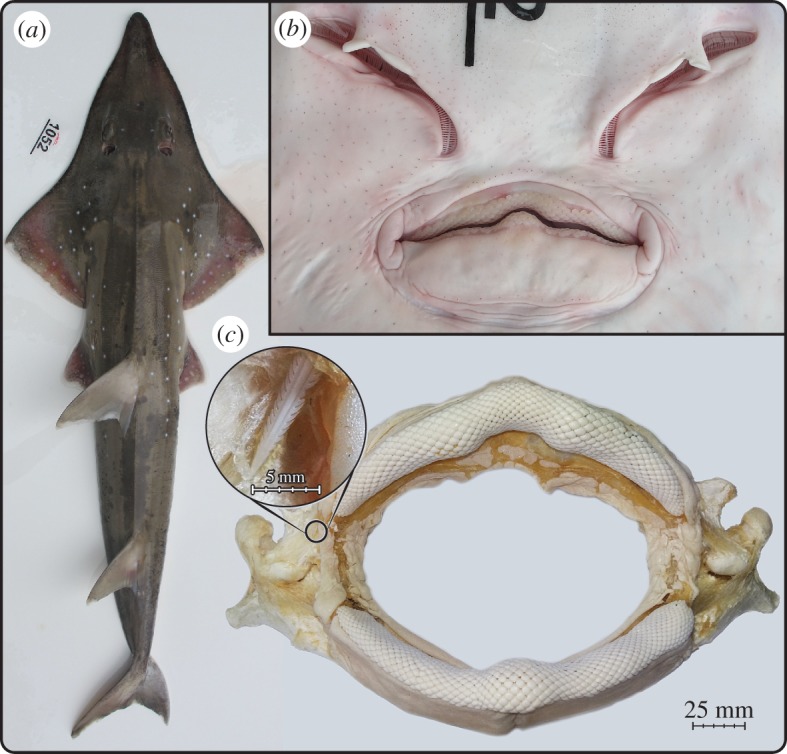
The wedgefish, *Rhynchobatus*. (*a*) Dorsal view of 115 cm TL female *R. australiae* from the South China Sea (ERB 1052). (*b*) Ventral view of the mouth of the same specimen. (*c*) The jaws investigated in this study (BMNH 2017.7.11.1), likely from an animal approximately 260 cm TL that was not reliably identified to species (see text). The inset shows the tip of a stingray spine that was visible externally, embedded in the soft tissue near the animal's upper jaw joint.

The material of interest is a dried jaw from a wedgefish (*Rhynchobatus* sp., BMNH 2017.7.11.1; figure 1*a*) collected in the Sula Sea, Philippines and purchased in 2015, presumably having been captured that year. The specimen was obtained from an artisanal fishery and was identified as being from *R. djiddensis*; however, a lack of additional biological material or documentation permit the possibility that it could instead be from *R. australiae*, given that both species are found in the capture area and reach the sizes we estimate for this individual (see Results). The jaw was probably whitened and sterilized with hydrogen peroxide and air-dried. It is largely skinned and defleshed, with only a thin covering of connective tissue (perichondrium) overlying most of the jaw cartilages, with additional layers of skin and muscle near the jaw articulation (figure 1*c*).

The jaw was photographed with a Canon EOS 1100D camera and then scanned with a Nikon Metris X-Tek HMX ST 225 XCT scanner at the Imaging and Analysis Centre, Natural History Museum, London. Scans were performed with a 0.25 mm copper filter at 190 kV source voltage and 160 µA source current over 360° sample rotation, with a final isotropic resolution of 84.14 µm voxel^−1^. The jaw exceeded the length of the scannable area; therefore, left and right sides were scanned separately and stitched together digitally using the Landmark Warp module in Amira ZIB Edition software (v. 2017.02; Zuse Institute Berlin, Germany). Several spines were embedded in the mineralized tissue of the jaw; to more closely characterize these tissue interactions, two sections, several centimetres long each and bearing embedded spines, were excised from the jaw and rescanned with higher resolution (29.24 and 31.63 µm voxel^−1^).

Scan data were investigated in two-dimensional slices and three-dimensional volumetric reconstructions, with the stingray spines manually segmented (digitally isolated) and all renderings and measurements performed in Amira ZIB Edition software and Avizo Standard software (v. 8.0.1; https://www.fei.com/software/amira-avizo/). In addition to the spines discussed below, a variety of radio-dense objects of varying size and shape were observed embedded in the tissue around the jaw joints; these were difficult to identify due to their small size, but most were apparently either tiny fragments of spines or sediment embedded in the tissue. Only those elements longer than 2 mm with clear pointed spine morphology were segmented and measured using the Label Analysis module in Amira ZIB Edition.

## 3. Results

**Figure 2.**
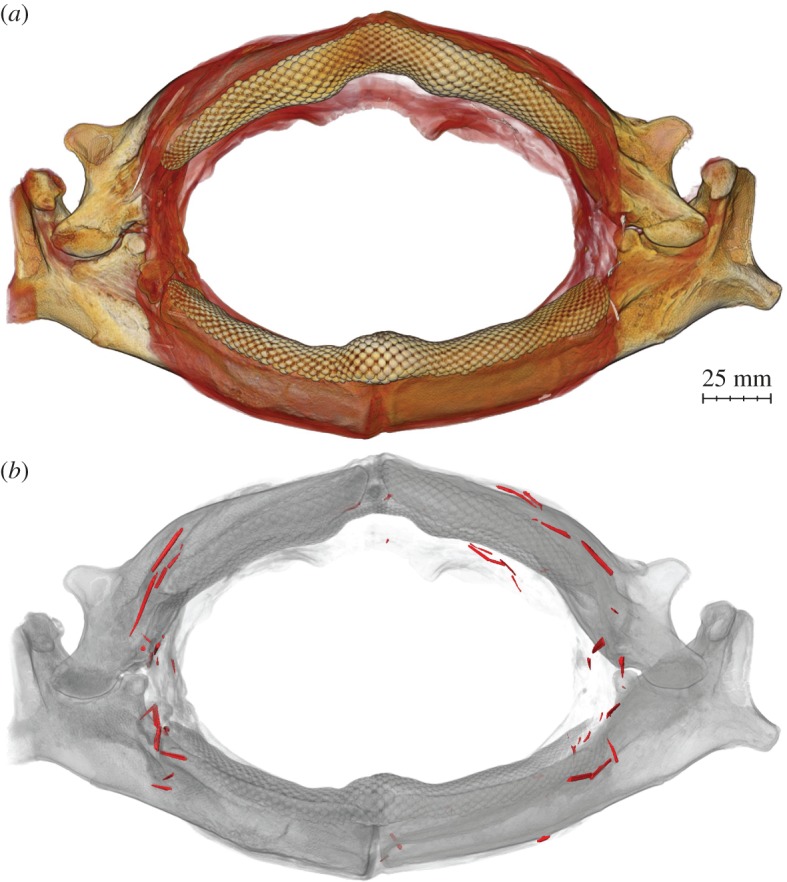
Volume renderings of micro-CT scans of the jaw in figure 1*c*. (*a*) Soft tissue is pseudo-coloured in red, teeth and mineralized jaw tissue (tesserae) are yellow; the numerous embedded stingray spines are white, but are largely obscured by soft tissue. (*b*) The jaw is rendered partially transparent and the embedded objects are coloured red. We identified 62 spine segments longer than 2 mm, the majority from stingray spines. See electronic supplementary material for an annotated video showing details of this scan.

The jaw is approximately 28.5 cm wide, and we estimate that the width of the animal's gape was approximately 14.5 cm (approx. 17.5 cm without soft tissue; figures 1 and 2*a*). There is no information available on the animal's size and maturity; however, using CT scans of a *R. djiddensis* specimen (LACM 38116-24) from a previous study [31], we measured the gape and jaw widths as approximately 7 and approximately 11%, respectively, of the animal's 76 cm total length (TL). This was similar to the gape/TL ratio for the *R. australiae* shown in figure 1. From this relationship, we estimate that the jaw likely came from an animal approximately 260 cm long, well above the reported size at maturity for either *R. australiae* [32] or *R. djiddensis* [33].

**Figure 3.**
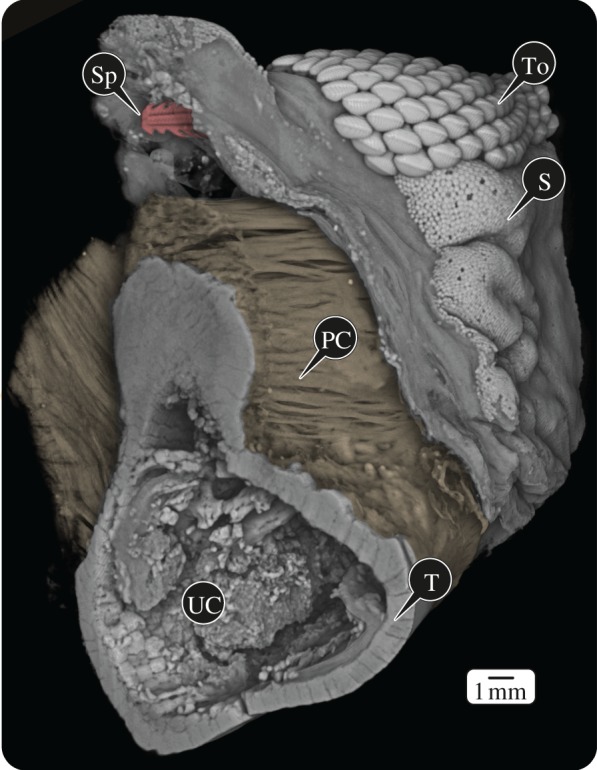
Volume rendering of a high-resolution scan of an excised portion of the jaw, looking at one of the cut ends. The majority of stingray spines were found embedded in either the subcutaneous layers of the skin (*S*) or the fibrous perichondrium (PC) wrapping the skeleton, which comprised an outer layer of mineralized tiles (tesserae, T) and an inner core of uncalcified cartilage (UC, collapsed here due to dehydration). A tooth series (To) is visible in the upper right and a broken stingray spine (Sp) in the upper left. In all regions of the jaws, the skin and soft tissue appeared undamaged, despite the spines and calluses we found beneath them (cf. figure 5*a*); this observation suggests that, in contrast to the skeletal tissue, perichondrium and dermis are capable of repairing wounds.

**Figure 4.**
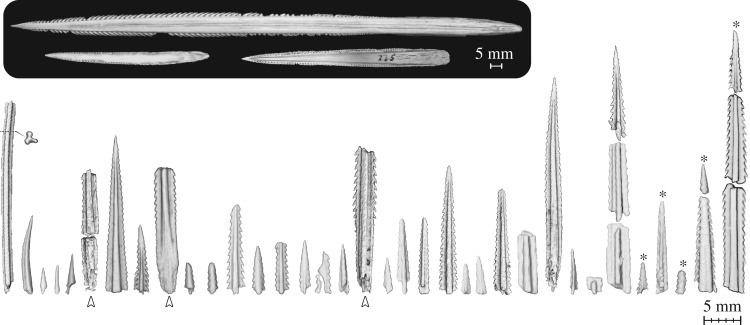
Volume renderings of spine elements (greater than 2 mm long) segmented from the jaw, with photos of intact stingray spines (inset) for comparison. Nearly all stingray spine elements were substantially damaged; however, in several cases, spines could be partially reassembled, based on proximity and fracture morphology. Spine elements marked with asterisks were encased in mineralized calluses on the surface of tesserae (figure 5). Spine elements marked with arrowheads beneath them are spine bases, evident by the lack of serrations and gradual taper towards their blunt, proximal ends. Numerous embedded elements could not be attributed to stingrays. For example, several spines similar to the one on the far left were observed, but could not be identified; these were long, non-tapered and had a ‘tripodal’ cross section (shown in cross-sectional view to right of spine). We also observed two spines like the second spine from the left, which has a shape and size suggestive of a squalid shark dorsal spine. Intact spines clockwise from top: *Brevitrygon walga* (BMNH 2017.7.14.2); *Potamotrygon* sp. (BMNH 2017.7.14.1); *Rhinoptera bonasus* (C. Underwood, personal collection).

Three-dimensional volume rendering of the *Rhynchobatus* jaw revealed 62 individual (radio-dense) objects longer than 2 mm, embedded predominantly in the soft tissues surrounding the mouth (figures 2 and 3). Objects were associated with both the labial and lingual faces of the jaws, but were clustered mostly around the corners of the mouth on the labial side. Approximately two-thirds of the embedded objects are unquestionably spines from stingrays, rather than from catfish or other spined marine animals, as evidenced by the recurved barbules on the lateral margins of the spines and their distinctive ‘mushroom’-shaped cross sections (figures 2 and 3; e.g. [34–36]). The remaining objects could not be identified unambiguously from CT scan data: most bear some resemblance to segments of bony fish dorsal fin spines, but two elements (approx. 3 and approx. 11 mm long) have sizes and shapes suggestive of squalid shark dorsal fin spines (figure 4) [37,38].

The considerable breakage of embedded spines precluded a definitive count of the total number of separate spines that originally impaled the animal (figure 4). In our CT reconstructions, it was also often difficult to determine whether pieces in close association originally belonged to the same spine. However, in several cases, segments found positioned end-to-end obviously originally belonged to and could be reassembled into single spines (figure 4), suggesting the spines fractured on impact. We estimate that 25–30 individual stingray spines are embedded in the jaw; however, it is also possible that additional stingray spines were removed and lost during the extraction and defleshing of the jaw.

The embedded stingray spine segments (including those reassembled from several pieces) ranged from 3.4–35.2 mm long (mean: 13.8 ± 8.8 mm) to 1.5–3.7 mm wide (mean: 2.5 ± 0.8 mm) and were predominantly the distal ends of spines, although several spine bases were observed (figure 4). Despite spine segments being different widths, comparable spine portions (i.e. tips or mid-shafts) were similar in shape, suggesting the gross morphology of intact spines would have been similar. In some cases, lateral barbules were not visible in scans; it is unclear whether this apparent lack was a function of scan resolution, the barbules having been worn down/broken as they entered the jaw tissue or the barbules having been resorbed after the fact (although the latter is unlikely; see Discussion).

**Figure 5.**
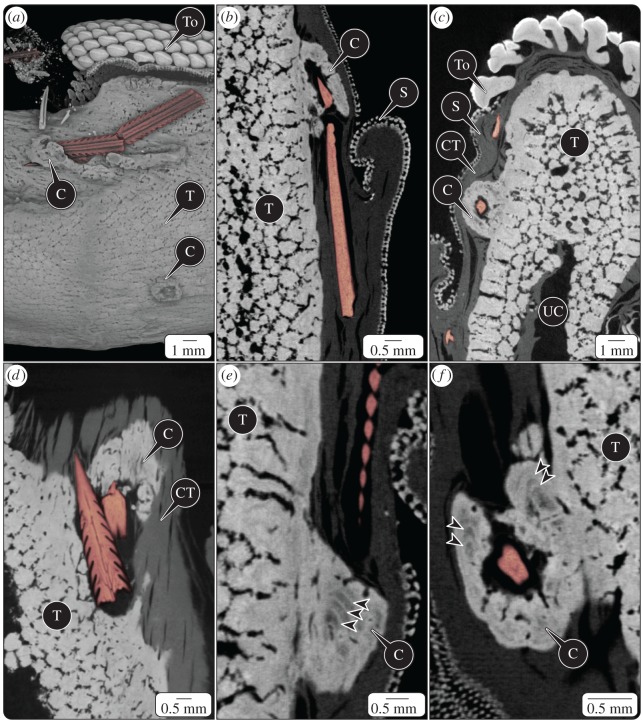
Callus formation associated with stingray spines (coloured red), embedded in the *Rhynchobatus* jaw. (*a*) Volume rendering of the jaw segment shown in figure 3 with most soft tissue removed; the spine in figure 3 is visible in the upper left corner in the background. The fractured spine in the middle of the image is partially encased in a trough-like callus (C). Calluses had an external morphology that clearly differed from that of tesserae (T) and could be identified, even in cases where the spine was no longer present (note the empty callus at the bottom of (*a*)). (*b–f*) Micro-CT sections through calluses. Note the callus Liesegang lines marked by arrowheads, the black gap between the callus and embedded spines, and the different morphology and apparent lower mineral density (greyscale value) of callus tissue relative to tesserae. Note also the numerous tesseral layers in the jaw (particularly beneath the tooth series (To) in (*c*)) and the additional spine elements embedded in the connective tissue (CT) beneath the skin (S); the perichondrium is the CT most closely associated with tesserae, but the borders between perichondrium and other connective tissues (e.g. dermis) are difficult to determine in these scans. The cavity marked by UC would have been filled with uncalcified cartilage in the hydrated jaw.

In several cases, spines were embedded, typically at their distal ends, into the mineralized tissue of the jaw (figures 4 and 5). In sharks and rays, the skeleton is largely comprised an unmineralized cartilage core, overlain by an outer rind of mineralized tiles called tesserae (figures 3 and 5), typically arrayed into a layer one or perhaps two tesserae thick [39–42]. The cortex of the jaw is modified to be multiple tesserae thick in species that experience high loads (e.g. due to a durophagous diet) and/or attain large body sizes (reviewed in [39]). The examined *Rhynchobatus* jaw exhibited a variable number of cortical layers of tesserae, depending on the location of the cross section, from two layers in flatter regions to more than 10 in curved regions (e.g. beneath the teeth or at the caudal ends of the jaws; figures 3 and 5). The outer tesseral layer typically exhibited taller, more ‘columnar’ tesserae [42].

Where we observed stingray spines embedded into jaw tissue, they apparently had not penetrated the tesseral layer, but rather mineralized masses had grown to encompass them (figure 5). We refer to these mineralized masses as ‘calluses’ as they represent a thickening and hardening of the tissue, but they should not necessarily be considered homologous to bone fracture calluses [43] (see Discussion). The tessellated cartilage calluses observed in this study were inconsistent in their structure, in some cases lacking organization (figure 5*c,d*), in others bearing texture or periodicity vaguely similar to tesserae (figure 5*b,f*) and in others lacking tessellation but bearing patterns like the Liesegang bands believed to be records of accretive growth in tesserae (figure 5*e,f*; reviewed in [41]). Calluses appeared to be anchored into the outer layer of columnar tesserae of the jaw, but exhibited lower or at least more variable mineral density/radio-opacity than adjacent tesserae (figure 5*b,c,f*). There was typically a dark/radio-lucent gap surrounding spines in their calluses (figure 5). Calluses never completely encased spines: the original insertion path of the spine was always clear, even in cases where embedded spine tips were broken away from the remainder of their spines (e.g. figure 5*b,d,f*). The ultrastructure of calluses and their internal cavities was distinct enough to allow their identification, even in two cases where previously embedded spines no longer were present (figure 5*a*).

## 4. Discussion

The presence of so many stingray spines in the jaw tissue of this *Rhynchobatus* specimen, the location of the spines in tissue on both the labial and lingual sides of the jaw and around the jaw joint (where batoids often clamp food during feeding [44,45]), the fact that some spines appear to have been embedded for a long period of time, and the similar appearance of jaw specimens from sharks known to feed on stingrays (e.g. [23]) all argue that this wedgefish was preying on stingrays with some regularity. It is impossible to identify the species of stingray involved, given the damaged state of many of the spines and the fact that stingray spines from different species or genera can be quite similar in appearance [35,46,47]. The shape and serrations of the observed spines, however, are less reminiscent of gymnurid or aetobatid ray spines than those of urobatid, myliobatid or dasyatid stingrays [34–36,48,49]. Predation on these stingray groups also is suggested by the species composition of stingrays in the region where the *Rhynchobatus* specimen was collected, and by our estimates for the maximum intact lengths of embedded spines (greater than 3 cm) [35,46,47,50].

The indication of frequent stingray predation by *Rhynchobatus* is surprising, given that its pebble-like dentition is more suggestive of a durophagous diet (figure 1). Our hypothesis of opportunistic piscivory by otherwise durophagous elasmobranch species is supported, however, by a single stomach content study showing that *Rhynchobatus* feeds on fish as well as hard-shelled prey [51] and by the presence of soft-bodied prey in the diet of adult cownose rays *Rhinoptera bonasus*, a species with flat, pavement-like teeth [19]. This disconnect between tooth form and function argues for vertebrate tooth morphology being a potentially unreliable predictor of diet (as with the villiform ‘snail-punching’ teeth of *Asemichthys taylori* [52]), while also suggesting that traditional sampling and analytical methods may be strongly biasing our perspectives on vertebrate feeding ecology, especially for larger individuals and species.

Although there are many piscivorous batoid species, predation on large fish is a difficult proposition for members of this clade. Batoid species are generally smaller than sharks, many are gape-limited, and few have teeth with the sharp cutting edges necessary to reduce large, soft-bodied prey into smaller pieces [20,21,25,31,45,50,53]. Batoid gapes—which are often both narrow and short—therefore can constrain the fish prey available to batoid species. As a result, prey approaching maximum gape size are ingested whole and on the shorter body axis: because large fish prey may exceed the length of the upper alimentary canal, it is not uncommon for piscivorous rays (e.g. skates, torpedo rays, butterfly rays) to be collected with an intact fish extruding from their mouths (J.J. Bizzarro 1999, 2003–2009, personal observation). The batoid gape constraint, therefore, structures the trophic ecology of non-durophagous species, perhaps explaining in part why stingrays, with their broad oblate body morphologies, are not known to be common prey.

Our observations suggest, however, that the potential limitations imposed by both anatomical gape and tooth shape can be overcome simply through the attainment of large body (and therefore gape) sizes. This conclusion challenges long-standing beliefs that ‘crushing-type’ dentition is synonymous with specialization on hard foods (e.g. [1,3,6]), that batoids do not consume stingrays [23,24], and that the diets of guitarfishes are largely restricted to crustacean prey (e.g. [50,54]). The primary reasons for these historical beliefs are sampling limitations and biases. Until recently, evaluations of elasmobranch diets were based largely on qualitative assessments of a limited number of stomach samples, with most being observations of relatively small individuals because of ease of capture and availability. Since piscivory is positively correlated with size for predatory shark and batoid species [25], most of the large specimens that are highly piscivorous are historically undersampled, often substantially. Furthermore, because most industrialized fishing nations are located in mid-latitudes, batoids occurring in temperate waters (e.g. skates) are relatively better studied than those in the tropics (e.g. stingrays, guitarfishes) [50].

These sampling issues have surely shaped the prevailing conceptions of the frequency and magnitude of intraclade predation within the elasmobranchs and the feeding behaviour and trophic spectrum of batoids. According to current dietary data, intraclade predation is relatively common in sharks (approx. one-third of 149 studied species [55]); however, only 10% of species have been found to have diets containing greater than 10% elasmobranchs, and no species' overall diet is dominated by (containing more than 50%) elasmobranchs [55]. Skates consume other elasmobranchs at almost the same frequency as sharks, but in lesser amounts [56]. Among 60 skate species, only one of the largest species had a diet consisting of greater than 10% elasmobranchs (*Dipturus nidarosiensis*, maximum TL = 230 cm [56]). Among 75 species of torpedo rays and stingrays, only 9% consumed elasmobranchs, and the greatest dietary contribution was only 2.4% (*Torpedo torpedo* [54]). However, recent meta-analyses have indicated that predation of elasmobranchs by batoids is more common than historically believed [57,58].

Our finding that some large batoids consume stingrays, therefore, disagrees with historic dogma. We argue, however, that, due to the aforementioned sampling limitations, it is likely that the frequency and especially the magnitude of predation within the elasmobranchs are under-reported, and that our results support an emerging concept of dietary plasticity in elasmobranchs. Investigations of individual dietary variability in elasmobranchs, and marine fishes in general, were virtually non-existent until recently, because data were routinely pooled for all members of a study population and individual feeding behaviour therefore was not evaluated [59,60]. Recent findings have demonstrated highly variable diets among sharks and batoids, mainly driven by spatial (location, depth) and temporal (diel, seasonal) factors and/or predator size [25,26]. In skates (the most studied and speciose batoid group), a strong link between predator size and prey size has been observed [27,56], with relatively small species consuming small crustaceans and polychaetes, medium-sized species eating crustaceans and small fishes, and large skates mainly eating larger fishes and decapods [25]. Furthermore, substantial individual dietary variability, including pronounced differences in diet among individuals of the same population, has also been well documented in skates [27–30].

**Figure 6.**
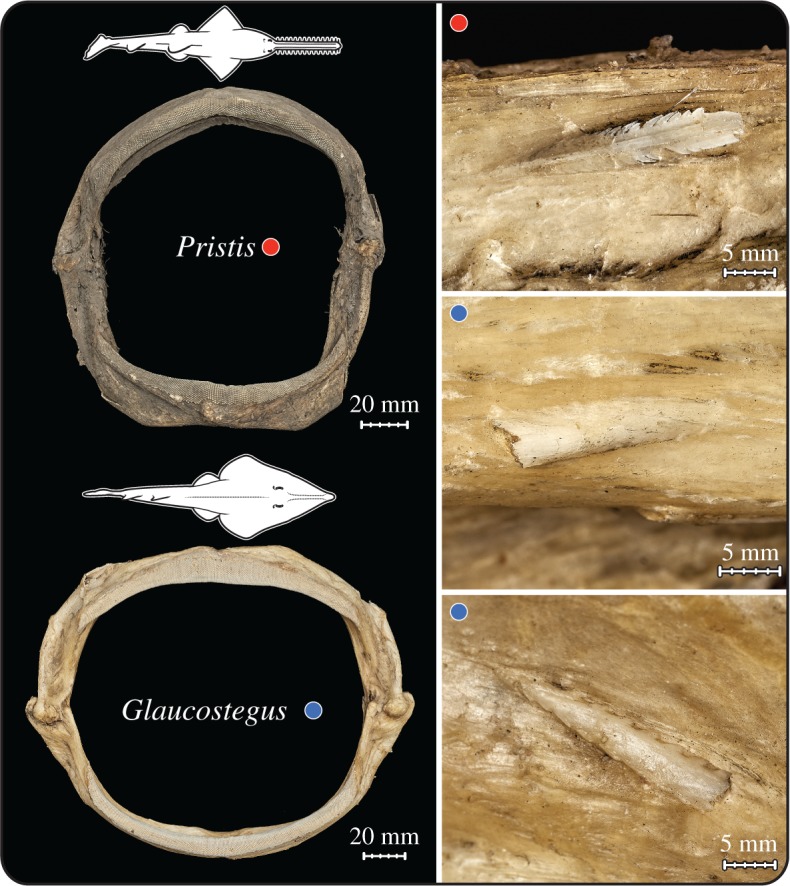
Dried jaws from two large batoids, the sawfish *Pristis* sp*.* (BMNH 2017.4.25.1) and guitarfish *Glaucostegus granulatus* (BMNH 1849.7.27.30). During external examination several spines were visible embedded in the soft tissues of the jaws (*Pristis* sp*.*, *n* = 15; *G. granulatus*, *n* = 20), as depicted in the magnified panels to the right. Collection data are lacking for the *Pristis* and *Glaucostegus* specimens, but size extrapolations suggest that both were large adults (greater than 2 m). Images copyright, Trustees of the Natural History Museum, London.

We argue that batoid predation on stingrays, though apparently only once previously reported [23], argues in favour of a high level of plasticity in batoid trophic ecology. Wedgefishes like *Rhynchobatus* are a sister group to the skates within the Rajiformes and appear to assume a trophic function in tropical environments that is similar to that of large skates in temperate and boreal regions. The stingrays of the Philippines, where our wedgefish specimen was collected, are diverse and abundant; therefore, stingrays are probably frequently encountered by wedgefishes sharing the same habitats [50]. The presence of stingrays in the diet of this *Rhynchobatus* may represent an individual dietary predilection, but we posit it is instead indicative of a more general but currently unappreciated phenomenon of opportunistic generalism in batoids, particularly in larger individuals. Upon visual inspection, we readily found an abundance of spines embedded in the dried jaws from two other large batoid specimens in the Natural History Museum collection: 15 spines in a jaw from a sawfish *Pristis* sp*.* and 20 spines in the jaw of a guitarfish *Glaucostegus granulatus* (figure 6). These counts likely underestimate the total number of spines embedded in the jaw tissues, as only five embedded spines were visible externally in the examined *Rhynchobatus* jaw. The presence of so many embedded spines in these three jaws argues that stingrays may be a common but largely undocumented food source for predatory batoid species that reach relatively large maximum sizes and thereby are released from the anatomical constraints that limit the diet of smaller individuals and species.

Prey defences often co-evolve with the behavioural and morphological adaptations of predators, and spines and other poisonous and/or stinging defences are typically believed to be major deterrents of predation (e.g. [61–65]). Stingray spines are formidable weapons: they can be more than 20 cm long in some species [46,47] (e.g. inset photo, figure 4) and the tissue trauma caused by their venom and puncture can be severe, long-lasting and occasionally fatal to potential predators and unwitting humans alike (even Odysseus was killed by a spear tipped with a stingray spine) (e.g. [66–69]). Stingray spines are apparently, however, ineffective as impediments to predation by large elasmobranch fishes. In addition to our evidence for stingray consumption by large batoids, large predatory sharks notoriously attack and eat stingrays, with jaws from some species occasionally exhibiting huge numbers of spines lodged in their tissues [23,24,48,70]. Our findings indicate, however, that impalement and envenomation from stingray spines clearly exerts a cost to predators in the form of damaged skeletal tissues.

Knowledge of how skeletal tissues respond to damage is vital to understanding how animals grow and meet mechanical and ecological challenges. Tessellated cartilage has been a defining character of shark and ray skeletons for hundreds of millions of years and comprises the vast majority of the skeleton, excepting only the vertebral centra, which are formed by so-called areolar mineralized tissue [39,42]. Until now, however, the mechanism by which shark and ray mineralized tissues react to injury has been unknown. Shark and ray cartilage, similar to mammalian cartilage but unlike bone, was thought to lack the ability to repair itself and to return damaged tissue to its original morphology (reviewed in [39,41]). This belief is based largely on data from a single laboratory experiment that induced damage to the skeleton and tracked the unmineralized tissue response [71] and on anecdotal observations of disorganized, naturally occurring mineralized calluses associated with previously damaged regions of the vertebral centra (areolar mineralized tissue; reviewed in [39]). It has also been suggested that elasmobranchs are incapable of resorbing foreign biological tissues, based on anecdotal observations of stingray spines embedded in sharks’ jaws [23] and studies surgically implanting bone particles in body soft tissues (subcutaneously or intramuscularly) [72,73].

Our findings provide the first unambiguous demonstration of a natural response of elasmobranch tessellated cartilage to injury. These observations lend credence to previous assertions that the elasmobranch skeleton is limited in its ability to repair and remove damage, with the structure of the observed calluses offering a window into their formation. Although calluses, like tesserae, appear to grow via accretion of mineralized tissue (as evidenced by Liesegang line patterning in some calluses; figure 5), the visible differences between tesserae and calluses in terms of morphology, ultrastructure and mineral density suggest that the mechanisms behind their formation and/or the underlying tissues on which they are formed may differ. Based on the relatively seamless connection between calluses and the outer portions of tesserae—which are patterned on collagen from the skeleton's outer fibrous perichondrium layer (‘PC’ in figure 3 and ‘CT’ in figure 5*c,d*) [74]—we posit that calluses are formed by tissue damage inducing mineralization of the perichondrium. The perichondrium is the only portion of the tessellated cartilage skeleton with appreciable vasculature or innervation and so is a likely source of callus-building cells, as in the periosteum of mammals [43,75]. The numerous calluses we identified in this single specimen and their similarity to the ‘cysts’ Gudger [24] noted encasing stingray spines embedded in the jaw of a hammerhead shark argue that calluses may be a common tissue response in elasmobranchs. Further study is needed to determine whether the pathways involved in the elasmobranch callus response are related to (e.g. truncated versions of) those that build mammalian bone calluses or foreign body granulomas [76], or instead alternative strategies evolved in this clade for responding to skeletal damage.

## Data accessibility

Renderings of the CT scan data are provided in an annotated video in the electronic supplementary material.
